# Osteitis Fibrosa Cystica: An Unusual Cause of Left‐Sided Chest Pain

**DOI:** 10.1155/crin/3576066

**Published:** 2026-01-08

**Authors:** Raahima Habib, Maha Anjum, Ayesha Mahmood, Nabiha Rizvi

**Affiliations:** ^1^ Department of Nephrology, Ghurki Trust and Teaching Hospital, Jallo Mor, Lahore, Pakistan

## Abstract

We report a case of osteitis fibrosa cystica resulting from secondary hyperparathyroidism in a 21‐year‐old male patient with end‐stage renal disease. The patient presented with persistent, moderate chest pain localized to the left fifth and sixth ribs for eight months. A chest X‐ray revealed well‐defined, expansile lytic lesions in these ribs. Prior testing showed elevated parathyroid hormone (PTH) levels for five years, along with decreased serum calcium and elevated phosphorus levels. Findings from ultrasound and SPECT scans were consistent with hyperparathyroidism. Repeat laboratory tests showed a PTH level of 939.8 pg/mL (normal: 10–69 pg/mL), calcium level of 8.3 mg/dL (normal: 8.4–10.2 mg/dL), and phosphorus level of 5.1 mg/dL (normal: 2.5–5.0 mg/dL). The patient declined surgical intervention and was managed conservatively with calcium and vitamin D supplementation. Within 4 weeks, symptoms resolved and calcium and phosphorus levels normalized, although PTH levels remained elevated. Osteitis fibrosa cystica can be challenging to diagnose due to its rarity, especially in developed countries, and its nonspecific clinical presentation. This case highlights the importance of considering this diagnosis and outlines an approach to management in resource‐limited settings.

## 1. Introduction

Secondary hyperparathyroidism is a common complication of chronic kidney disease (CKD), occurring in about 40%–80% in patients with Stage 3 and Stage 4 CKD, respectively [1]. As a result of renal dysfunction, serum levels of phosphorus increase, whilst calcium and calcitriol decrease, stimulating the parathyroid glands to secrete high levels of parathyroid hormone (PTH) [2]. A rare manifestation of untreated secondary hyperparathyroidism is osteitis fibrosa cystica (OFC), a non‐neoplastic osteolytic metabolic bone disease [3]. Clinically, it may present as fractures, skeletal deformities, and bone pain [4]. To prevent effects on quality of life, it is important to ensure early diagnosis and effective treatment.

We present the case of a 21‐year‐old male with end‐stage renal disease, presenting with persistent chest pain due to OFC. This case report will aid in understanding the disease since its symptoms are nonspecific and overlap with other conditions such as bone tumors [3]. Furthermore, this case highlights the challenges of management in resource‐constrained settings, where prevalence is higher [5].

## 2. Case Presentation

A 21‐year‐old male, a known case of end‐stage renal disease for the past six years and undergoing hemodialysis three times a week, presented to our renal outpatient department with left‐sided chest pain. Given the location of the pain and the patient’s comorbidities, ischemic heart disease was considered a possibility and needed to be ruled out due to its potential severity. However, further history revealed that the chest pain had been ongoing for eight months, was localized to the lateral aspect of the left fifth and sixth ribs, and did not radiate. The pain had a gradual onset, was moderate in intensity, and had persisted since its onset, even at rest. It was aggravated by movement but not affected by lying down. Initially, the pain responded to paracetamol and muscle relaxants, but it recurred after 2 months with increased intensity. There were no associated symptoms such as dyspnea, epigastric pain, or diaphoresis, which made cardiac causes less likely. Pulmonary causes were excluded due to the absence of respiratory symptoms, including cough, sputum production, hemoptysis, or fever. Costochondritis was considered in the differential diagnosis; however, it was deemed less likely due to the chronicity of the pain, its localization away from the costochondral joints, and its persistence at rest, which are atypical features for that condition. Notably, the patient had a history of a left femur fracture following a minor slip one and a half years ago, which was managed conservatively, suggestive of possible underlying bone fragility. There was no surgical history. There was no palpable mass on the chest and no evidence of bone deformities such as kyphosis or loss of height. Neck examination did not reveal any palpable masses.

**Figure 1:**
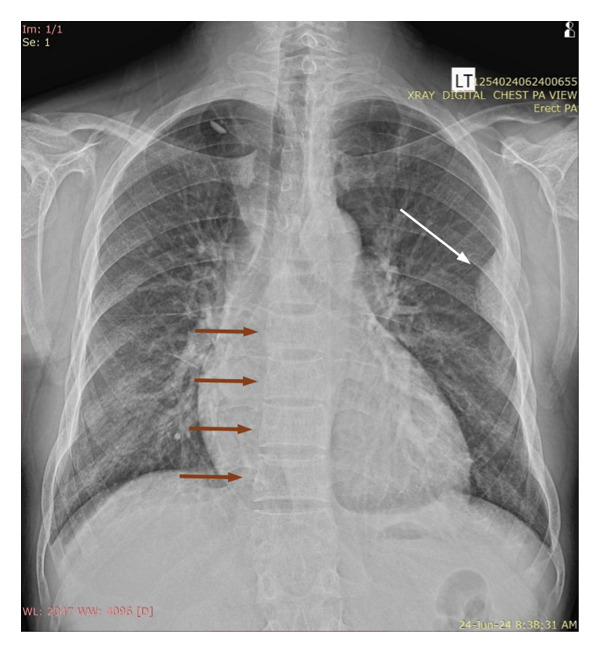
Chest X‐ray with expansile lytic lesion on outer border of fifth and sixth ribs (white arrow) and biconcave appearance of thoracic vertebral bodies (red arrows).

In view of the chronic chest pain and clinical suspicion, an electrocardiogram (ECG) and chest X‐ray were performed. The ECG was unremarkable. The chest X‐ray showed a well‐defined expansile lytic lesion on the outer border of the fifth and sixth ribs with overall decreased density of the skeleton (Figure 1). A biconcave appearance was also observed in the thoracic vertebral bodies, a feature consistent with metabolic bone disease. The X‐ray was followed by a high‐resolution CT scan of the chest (Figure 2). A diffuse patchy sclerotic density was appreciated in visualized bones with multifocal expansile bony lesions in ribs, especially on the left fifth rib and head of the right humerus. A small nodule in the left lobe of the thyroid was also noted. These findings were indicative of hyperparathyroidism.

Figure 2CT chest with diffuse patchy sclerotic density in visualized bones with multifocal expansile bony lesions in ribs (black and white arrows) and head of right humerus without cortical break (red arrow).

**Figure:**
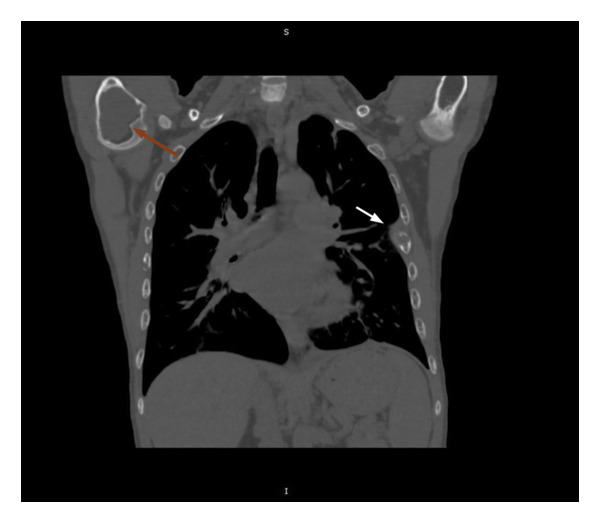
(a)

**Figure:**
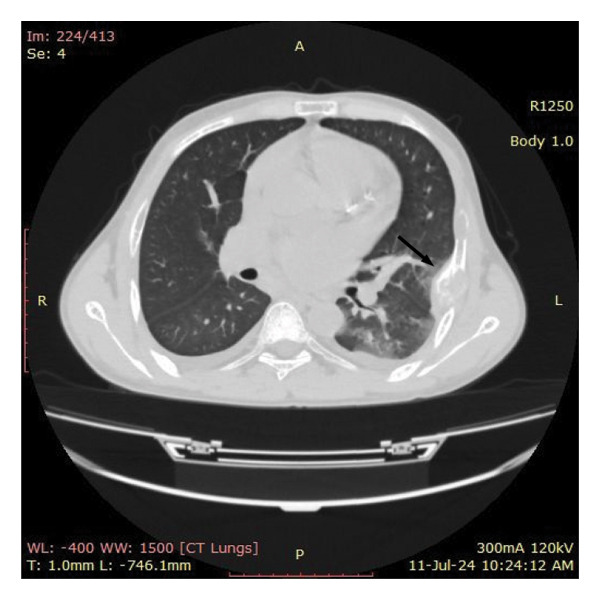
(b)

**Figure:**
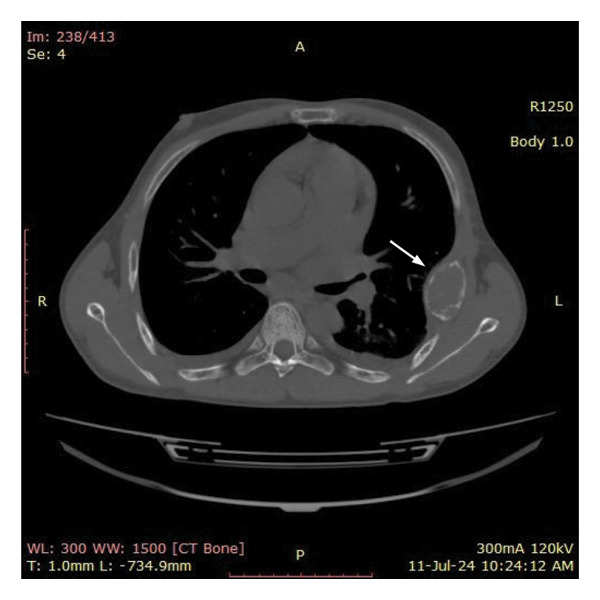
(c)

On further inquiry, the patient revealed that he had a workup for secondary hyperparathyroidism before. His reports revealed persistently elevated PTH for five years (from 2018 to 2023). There were also increased levels of serum phosphorus and decreased levels of serum calcium. The values in chronological order are presented in Table 1. A prior thyroid ultrasound and single‐photon emission computed tomography (SPECT) scan were also performed. The ultrasound showed a hypoechoic nodule posterior to the left lobe of the thyroid. The SPECT scan images initially demonstrated homogenous radiotracer uptake in both lobes of the thyroid gland and two areas of focal radiotracer uptake just inferior to the lower poles of thyroid lobes bilaterally. On delayed images, there was a washout of radiotracer activity from the thyroid gland and retention of focal tracer uptake just inferior to the lower poles of thyroid lobes bilaterally. On correlative CT, two well‐defined soft tissue nodules were present in these areas. These findings were consistent with hyperfunctioning parathyroid glands.

**Table 1:** Lab values of serum calcium, serum phosphorus, and PTH in chronological order.

	Serum calcium (mg/dL)	Serum phosphorus (mg/dL)	PTH^∗^ (pg/mL)
Normal range	8.4–10.2	2.5–5.0	10–69
Date (MM/YY)			
01/18	8.9	9.6	
01/22	7.4	6.0	3512
02/22	8.2	4.4	2709
04/22	7.4	5.2	2505
05/22	6.5	6.9	3339
06/22	7.4	5.8	3309
06/22	7.5	5	2693
11/22			2313
05/23	8.2	6.4	
10/23	8.4	3.9	
07/24	8.3	5.1	939

^∗^PTH: parathyroid hormone.

The lab tests were then repeated to obtain fresh results. PTH levels came out to be 939.8 pg/mL (normal: 10–69 pg/mL), calcium levels were 8.3 mg/dL (normal: 8.4–10.2 mg/dL), and phosphorus levels were 5.1 mg/dL (2.5–5.0 mg/dL).

He had been advised a parathyroidectomy previously but had refused due to financial constraints, and again opted out of it. The patient was prescribed calcitriol injections (1 μg) after every session of hemodialysis and oral vitamin D and calcium‐containing phosphate binders. In 4 weeks of starting treatment, the patient reported being pain‐free. His calcium and phosphorous levels had normalized (9.4 and 4.6 mg/dL, respectively). PTH levels, however, remained elevated at 1015 pg/mL.

## 3. Discussion

Secondary hyperparathyroidism is a consequence of CKD. Increased levels of serum phosphate, decreased serum ionized calcium, reduced serum calcitriol, and elevated levels of the phosphatonin Fibroblast growth factor 23 (FGF‐23) stimulate the parathyroid glands to synthesize and release PTH, which increases osteoclastic activity [6]. If left untreated, it can eventually lead to the development of OFC.

OFC is a rare manifestation of secondary hyperparathyroidism, due to optimal management of end‐stage renal disease and early detection [7, 8]. However, this is the case for more developed countries where quality treatment is available and screening occurs. In resource‐limited areas and among patients with a lower socioeconomic background, cases are still being reported. Unfortunately, bone lesions secondary to hyperparathyroidism tend to be diagnosed once the disease has already progressed to its advanced stages, when symptoms become apparent [4].

According to a study by Xie et al. (2019), brown tumors are usually located in a single site, with a preference for the mandible, clavicle, ribs, and pelvis; however, the frequency of these tumors in the skull and association with nasal and paranasal sinuses is lower. Radiological findings include well‐defined osteolytic lesions, usually with bone expansion, and can show bony destruction. This presentation, however, can vary. Ill‐defined lesions, mixed lytic/sclerotic lesions, or margins, with adjacent soft tissue involvement, may also occur [9]. The lack of specificity of these findings makes the lab findings of decreased calcium, increased phosphate, and PTH essential for making the diagnosis of renal OFC. Previous case reports have also demonstrated that it can mimic bone diseases, complicating diagnosis even further [3, 10].

Treatment of OFC aims to replace serum calcium and decrease phosphate levels. This includes dietary restriction of phosphate, phosphate binders, calcium, vitamin D supplementation, calcimimetics, and parathyroidectomy [5, 11].

Active vitamin D compounds increase the absorption of both calcium and phosphorus from the gut and reduce the synthesis of PTH. A narrow therapeutic index of vitamin D analogs, however, can limit its use. Pharmacological doses can result in hypercalcemia and hyperphosphatemia, which can lead to calcification of vessels [6].

Phosphate binders, such as sevelamer, are calcium‐free drugs that decrease phosphate levels. They do not have any effect on calcium levels and do not reduce PTH levels significantly, making their use as a preventative measure in early CKD more important [6].

PTH values greater than 800 pg/mL for more than 6 months that do not respond to medications are an indication of parathyroidectomy [10]. One report showed marked improvement of bone lesions in 7 weeks [12]. Despite this, our patient did not undergo surgery, due to limited financial resources, and was managed through pharmacological therapy only.

Those patients who cannot undergo surgery can benefit from long‐term administration of cinacalcet, which can reduce the size of the parathyroid gland. However, there is a significant increase in PTH, calcium, and alkaline phosphatase within 12 months of cinacalcet cessation, requiring continuous use. This can be a problem, since cinacalcet also induces gastrointestinal symptoms and drug–drug interactions, reducing patient compliance [13].

In this setting, the patient’s choice in treatment was influenced by affordability, making regular use of expensive medications, such as cinacalcet, and surgical management unfeasible. Instead, management with calcium and vitamin D supplementation was preferred.

In summary, although OFC is an uncommon presentation of hyperparathyroidism, it significantly affects the quality of life. This highlights the importance of early diagnosis and effective management. Our case study adds to the limited literature on OFC, aiding clinicians in recognizing the disease more effectively. Furthermore, there is a need to explore management strategies in low‐income countries, where its incidence is higher, and treatment options may be limited.

## Consent

Written informed consent was obtained from the patient for the publication of this study.

## Conflicts of Interest

The authors declare no conflicts of interest.

## Funding

No funding was obtained for this study.

## Data Availability

The data that support the findings of this case report are available from the corresponding author on reasonable request.
